# Ferromagnetism in Transitional Metal-Doped MoS_2_ Monolayer

**DOI:** 10.1186/s11671-016-1376-y

**Published:** 2016-03-22

**Authors:** Xiao-Li Fan, Yu-Rong An, Wen-Jun Guo

**Affiliations:** State Key Laboratory of Solidification Processing, School of Material Science and Engineering, Northwestern Polytechnical University, 127 YouYi Western Road, Xi’an, Shaanxi 710072 China; Beijing Computational Science Research Center, Beijing, 100094 China

**Keywords:** MoS_2_, Transitional metal, Doping, Magnetic interaction, First-principles calculations

## Abstract

Manipulating electronic and magnetic properties of two-dimensional (2D) transitional-metal dichalcogenides (TMDs) MX_2_ by doping has raised a lot of attention recently. By performing the first-principles calculations, we have investigated the structural, electronic, and magnetic properties of transitional metal (TM)-doped MoS_2_ at low and high impurity concentrations. Our calculation result indicates that the five elements of V-, Mn-, Fe-, Co-, and Cu-doped monolayer MoS_2_ at low impurity concentration all give rise to the good diluted magnetic semiconductors. By studying various configurations with different TM-TM separations, we found that the impurity atoms prefer to stay together in the nearest neighboring (NN) configuration, in which the doped TM atoms are FM coupling except for Fe doping at 12 % concentration. For V, Mn, and Fe doping, the total magnetic moment is smaller than the local magnetic moment of the dopants because the induced spins on the nearby host atoms are antiparallel to that of the doped atoms. In contrast, Co and Cu doping both give the higher total magnetic moment. Especially, Cu doping induces strong ferromagnetism relative to the local spins. However, the atomic structures of Co- and Cu-doped MoS_2_ deviate from the original prismatic configuration, and the magnetic moments of the doped systems decrease at 12 % impurity concentration although both elements give higher magnetic moments at 8 % impurity concentration. Our calculations indicate that V and Mn are promising candidates for engineering and manipulating the magnetism of the 2D TMDs.

**Electronic supplementary material:**

The online version of this article (doi:10.1186/s11671-016-1376-y) contains supplementary material, which is available to authorized users.

## Background

Research on two-dimensional (2D) transitional metal dichalcogenides (TMDs) has attracted considerable attention due to their distinct electronic, optical, and catalytic properties [1–5]. Group 6 TMDs (MX_2_, M = Mo, W, and X = S, Se, Te) hold promise for flexible and transparent electronics applications owing to their sizeable band gaps ranging from 1 to 2 eV. Current results have indeed revealed that MoS_2_ and WS_2_ form an exciting family of transistors [6–11]. On the other side, MoS_2_ and WS_2_ are nonmagnetic semiconductors. Accordingly, extensive studies have been performed to investigate the feasible ways to introduce magnetism to MoS_2_, such as morphology fabricating [12–14], external strain, [15–17] and impurity doping [18–28].

Developing approaches to effectively induce and manipulate magnetism are critical to the use of the magnetic nanostructures in quantum information devices. Among kinds of magnetic property engineering methods, doping attracts more attentions [18–28]. On the basis of previous studies, transitional metal (TM) atom doping can effectively induce magnetism into MoS_2_. For example, magnetism is observed for Mn [18, 20, 22, 24, 26, 28], Fe [18, 22, 24, 26, 28], Co [18, 22, 24, 26, 28, 29], Cr [18, 24], Zn [22, 24], Cd [24], and Hg [24] doping. And the magnetic moment of the 3*d* TM-doped MoS_2_ increases with the *d*-band filling of the TM dopants [18]. Additional, spin polarization was found in MoS_2_ with S atoms replaced by incomplete *d*-band atoms, such as Fe and V [30], and Group VA and III elements, such as N, P, As, B, Al, and Ga [28]. Moreover, adsorption of various atoms, such as H, B, C, N, and F, is also effective to turn MoS_2_ from nonmagnetic to magnetism [31]. It is worth noting that no magnetism is observed in V-doped MoS_2_ based on Ref [24], but according to Ref [18] and [28], V doping induces more than 1-μ_B_ magnetic moments into monolayer MoS_2_. And based on Lee’s study [22], the nonmagnetic element Cu doping brings strong magnetism into the doped MoS_2_.

More recently, substitutional doping MoS_2_ monolayer with magnetic atom and the interactions between the doped atoms has draw intensive attentions. Ramasubramaniam [20] have studied the Mn-doped monolayer MoS_2_ at concentration of 10–15 % by performing the density functional theory calculations and Monte Carlo simulations, which shows that the doped Mn atoms couple ferromagnetically. Schwingenschlögl et al. [24] predict that the doped TM atoms are ferromagnetic (FM) ordering for Mn, Zn, Cd, and Hg doping at 6.25 % impurity concentration and antiferromagnetic (AFM) ordering for Fe and Co doping. Similarly, Mishra et al. [26] predict the FM ordering in fairly diluted Mn doping MoS_2_, MoSe_2_, MoTe_2_, and WS_2_ and AFM coupling for Fe and Co doping at large separations. In contrast, a later study [18] found the ground states of Mn-, Fe-, and Co-doped MoS_2_ are all FM.

Clearly, current studies on the magnetic interactions in Mn-, Fe-, and Co-doped MoS_2_ disagree with each other. However, the magnetic ordering of the dopants as well as the orientations of the induced spins on the host atoms are critical factors to determine the magnetic property of the doped system. In this context, we examined the cases of different impurity concentrations and separations of the doped atoms to study the electronic and magnetic properties of TM-doped monolayer MoS_2_ and to find out the magnetic feature of the TM-doped 2D TMDs. Five 3*d* TM elements including V, Mn, Fe, Co, and Cu doping were studied in the present work by accurate calculations. Our calculations result indicates that the doped TM atoms prefer to stay in the nearest neighboring configurations and ferromagnetic coupling with each other. Additionally, we found that at high impurity concentrations, the local structures around the dopants were deformed from the original prismatic configurations. More importantly, it was found that V and Mn doping are the good candidate to induce and manipulate the magnetism into 2D TMDs, but Cu is not although it can induce strong magnetism.

## Methods

The first-principles calculations were carried out by using the Vienna ab initio simulation package (VASP) based on the density functional theory (DFT) [32]. The electron-ion interactions were described by the projector-augmented wave (PAW) method [33, 34]. The generalized gradient approximation of the Perdew-Burke-Ernzerhof (PBE-GGA) [35] formula was used for the electronic exchange-correlation potential. In addition, Hubbard-U parameterization method with a common U value of 3.0 eV was assigned to all the 3*d* impurities. The U parameterization was not used for the host materials since there little impact on the magnetic ordering [18, 26, 36]. The substitutional TM doping was calculated with a 5 × 5 × 1 supercell. A vacuum region of 15 Å was added to avoid interactions between adjacent images. The Brillouin zone was sampled by the Monkhorst-Pack method [37] with a 2 × 2 × 1 k-point grid. The wave functions were expanded in a plane wave basis with an energy cutoff of 600 eV. The convergence criterion for the self-consistency process was set to 10^−5^ eV between two ionic steps, and the convergence criteria of 0.02 eV/Å were adopted for total energy calculations.

## Results and Discussions

**Fig. 1:**
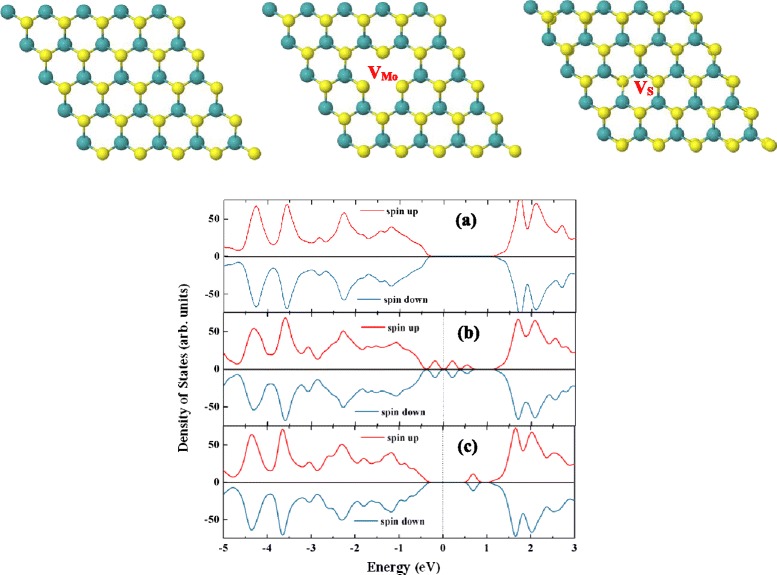
Top views and density of states of monolayer MoS_2_ without and with Mo/S vacancy. Density of states of monolayer MoS_2_ (*a*), monolayer MoS_2_ with Mo vacancy (*b*), monolayer MoS_2_ with S vacancy (*c*)

The fully relaxed lattice constants are *a* = *b* = 3.18 Å for single layer MoS_2_, and the distance between Mo and S atoms are 2.41 Å. Figure 1 shows the atomic structure and density of states for monolayer MoS_2_, which is nonmagnetic semiconductor. Our calculated band gap is 1.70 eV with the valence band maximum and conduction band minimum both locating at Κ point. Morphology fabricating such as atomic defects is a useful way to bring ferromagnetism into the low-dimensional materials [38, 39]. But according to previous study [17, 40], neither Mo vacancy nor S vacancy changes the nonmagnetic property of monolayer MoS_2_. Also, Si vacancy does not bring magnetism into silicene [41, 42]. As shown in Fig. 1, our calculation shows the same results.

**Fig. 2:**
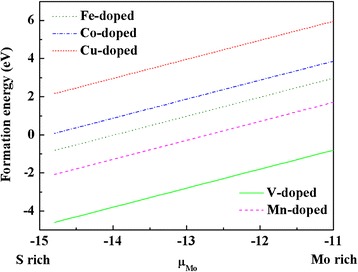
Formation energies for TM atom substitutional doping MoS_2_ as a function of chemical potential of Mo (μ_Mo_)

We firstly studied the doping at low impurity concentration. The doping concentration is defined as the number of doped TM atoms divides by the total number of Mo atoms. Hence, if one Mo atom is replaced by one TM atom in a 5 × 5 × 1 supercell, the corresponding impurity concentration should be 4 %. The formation energies of TM substitutional doping MoS_2_ are calculated via the following formula:

$$ {E}_f=E\left({\mathrm{TM}}_{\mathrm{Mo}},{\mathrm{Mo}\mathrm{S}}_2\right)-E\left({\mathrm{Mo}\mathrm{S}}_2\right)-{\mu}_{\mathrm{TM}}+{\mu}_{\mathrm{Mo}} $$

where *E*(TM_Mo_, MoS_2_) and *E*(MoS_2_) represent the total energies of MoS_2_ with and without TM doping, respectively. *μ*_TM_ is the chemical potential of a single-doped TM atom in its stable bulk lattice. For Mo-rich condition, *μ*_Mo_ is taken as the energy of a Mo atom in its stable fcc lattice and for S-rich condition *μ*_Mo_ is determined from the energy difference between a S_2_ molecule and one MoS_2_ unit. Figure 2 shows our calculated formation energies for TM doping MoS_2_ as a function of *μ*_Mo_. It shows that the V and Mn doping are favorable energetically, especially under S-rich growth conditions. The *C*_3*v*_ symmetry is destroyed after TM doping, and the distances between the doped TM and the nearest S atoms are 2.39, 2.36 2.44, 2.30, and 2.42 Å for V, Mn, Fe, Co and Cu doping.

**Fig. 3:**
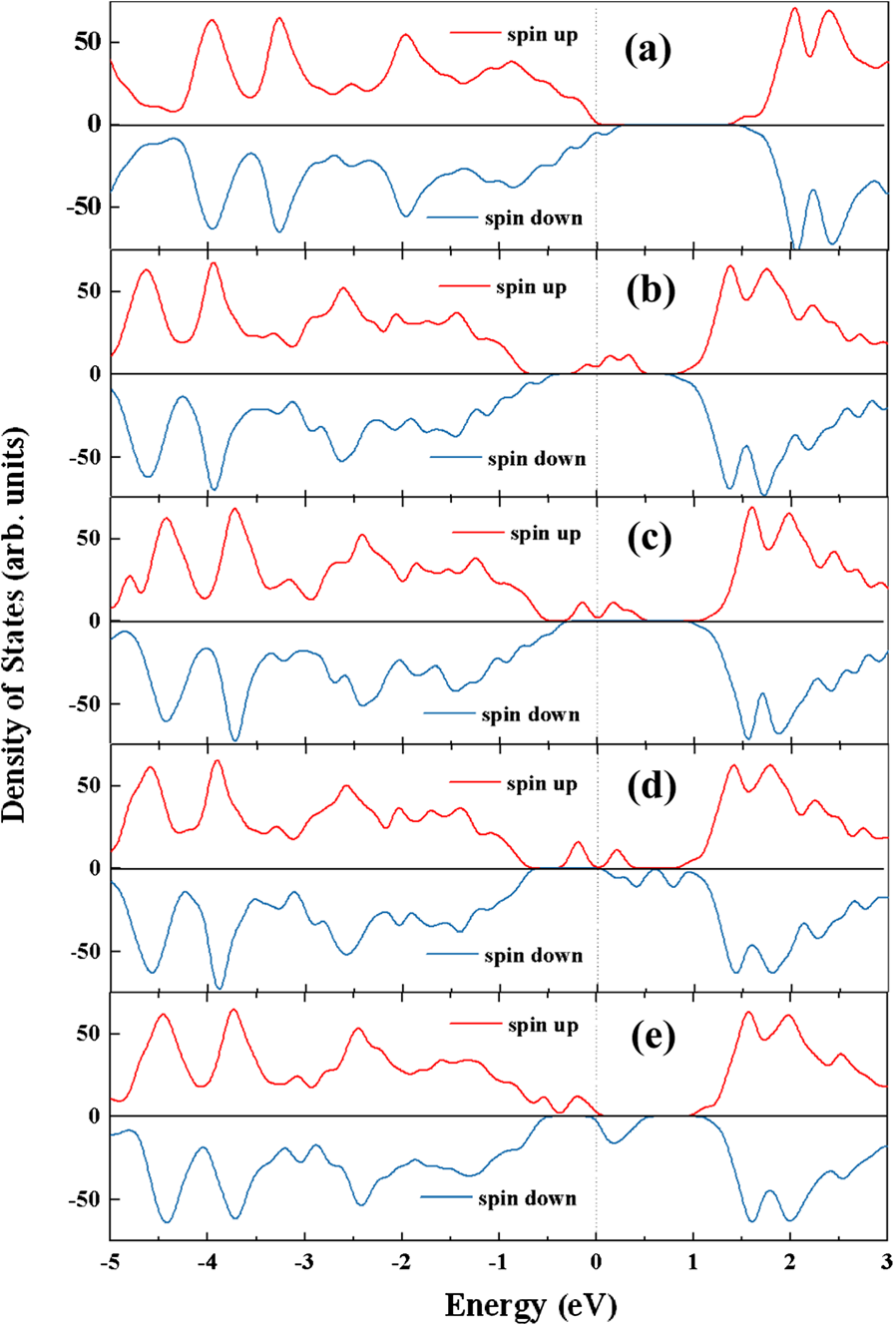
Density of states of monolayer MoS_2_ with V, Mn, Fe, Co, and Cu doping at 4 % impurity concentration. *a* V doping, *b* Mn doping, *c* Fe doping, *d* Co doping, *e* Cu doping

Figure 3 shows our calculated DOSs for TM-doped monolayer MoS_2_ at 4 % impurity concentration. As shown in Fig. 3, defect states appear within the band gap for all the doped systems and they are highly localized. The defect states are mainly contributed by the doped TM 3*d* states. Additionally, for the V-, Mn-, Fe-, and Cu-doped systems, both the defect states and Fermi level are more close to the valence band, but for the Co-doped system, the impurity level and Fermi level are more close to the conduction band. This is different from previous result by Lebruton et al. [28] Based on their DFT/PBE calculations, they have predicted that for Mn-, Fe-, and Co-doped MoS_2_, the impurity level and Fermi level both are more close to the conduction band. However, as shown in Additional file 1: Figure S1, our calculations result with no U parameterization which agrees with the results of Leburton et al. [28] and Lee et al. [22]. Moreover, all the doped systems are still semiconductor although the band gaps reduce a lot relative to the original value before doping.

More importantly, Fig. 3 clearly shows the induced spin polarization for all the five doped systems. The corresponding magnetic moments are 1, 1, 2, 3, and 4.9 μ_B_ for V, Mn, Fe, Co, and Cu doping, respectively. An isolated V atom has a 3*d*^4^4*s*^1^ electronic configuration with one valence electron less than Mo (4*d*^5^5*s*^1^), which reflects the magnetic moment of the V-doped MoS_2_. The electronic configurations of isolated Mn, Fe, Co, and Cu atom are 3*d*^5^4*s*^2^, 3*d*^6^4*s*^2^, 3*d*^7^4*s*^2^, and 3*d*^10^4*s*^1^, respectively; they have one, two, three, and five additional valence electrons compared to Mo atom, which consist with the magnetic moment of Mn-, Fe-, Co-, and Cu-doped system. As shown in Fig. 3, the spin splitting appears near to the Fermi level, which is contributed by the defect states associated with the doped TM atom, *p* states of the adjacent S atoms, and *d* states of the nearby Mo atoms. We further calculated the spin-resolved charge density to investigate the distribution of these magnetisms.

**Fig. 4:**
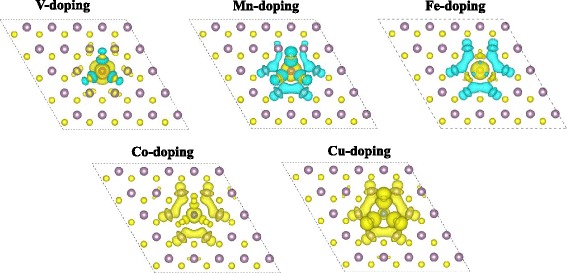
The spin-resolved charge density isosurface (isosurface value at 0.002 e/Å^3^) of TM-doped MoS_2_ monolayer at 4 % impurity concentration. *Yellow* and *blue colors* represent the spin-up and spin-down charges, respectively

As shown in Fig. 4, we can see the spin polarization localized on the dopants and the nearby S and Mo atoms, as well as the interstitial region. Strong hybridization between the TM 3*d* states and the *p* states of the adjacent S atoms yields spin splitting to the S atoms. For V and Mn doping, the spins of the dopants are antiparallel to the induced spin of the nearest three S atoms. In V-doped MoS_2_, the induced spins on the nearest six Mo atoms are parallel to that of the doped V atom; correspondingly, the total magnetic moment is little larger than the local magnetic moment of the dopant. For Mn doping, the induced spins on the nearest three S atoms and six Mo atoms are all antiparallel to the local spin on the impurity atom, which makes the total magnetic moment much smaller than the local magnetic moment of the dopant. Comparing to Mn doping, the induced spins on the three nearest S atoms are parallel to that of the Fe dopants. Plus, the local magnetic moment on Fe is larger than that on Mn. Consequently, the total magnetic moment of Fe-doped MoS_2_ is larger than that of the Mn-doped system. As for the Co and Cu doping, the induced spins on the nearest S and Mo atoms are all parallel to that of the doped TM, which give rise to the much larger total magnetic moment although the local magnetic moment on the dopants are small. Particularly, the local magnetic moment on the doped Cu is 0.5 μ_B_, which induces 0.2–0.3 μ_B_ ferromagnetism on the nearby S and Mo atoms, plus the interstitial region; the consequent total magnetic moment of doped system is 4.9 μ_B_.

In the last part, we have studied TM doping at 4 % impurity concentration by calculating one TM atom replacing one Mo atom in a 5 × 5 × 1 supercell, in which the distance between the dopants is around 16 Å. We further calculated two TM atoms replacing two Mo atoms in a 5 × 5 × 1 surpercell to investigate the TM doping at 8 % impurity concentration. There configurations with different TM-TM separations were considered: NN configurations in which the two TM atoms are in the nearest neighboring position with TM-TM distance of 3.2 Å, the second NN configurations in which the two TM atoms are in the next nearest-neighboring position with TM-TM distance of 5.5 Å, and the third NN configuration in which the distance between the two doped TM atoms are 6.5 Å.

**Fig. 5:**
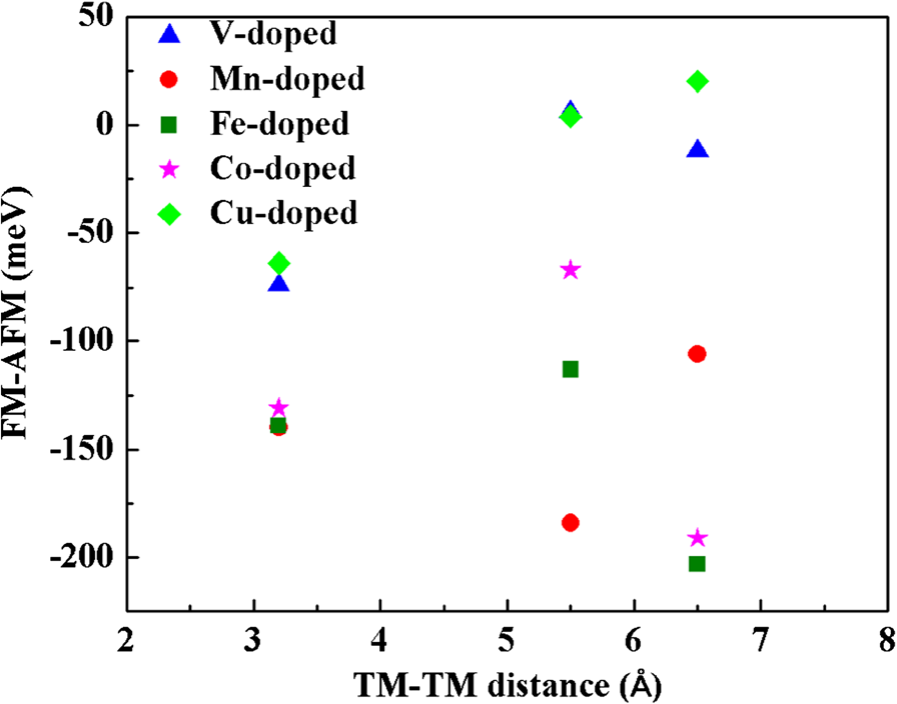
Energy differences of the FM ordering over AFM ordering for V-, Mn-, Fe-, Co-, and Cu-doped MoS_2_ as a function of the distance between the two doped TM atoms. The corresponding impurity concentration is 8 %

**Fig. 6:**
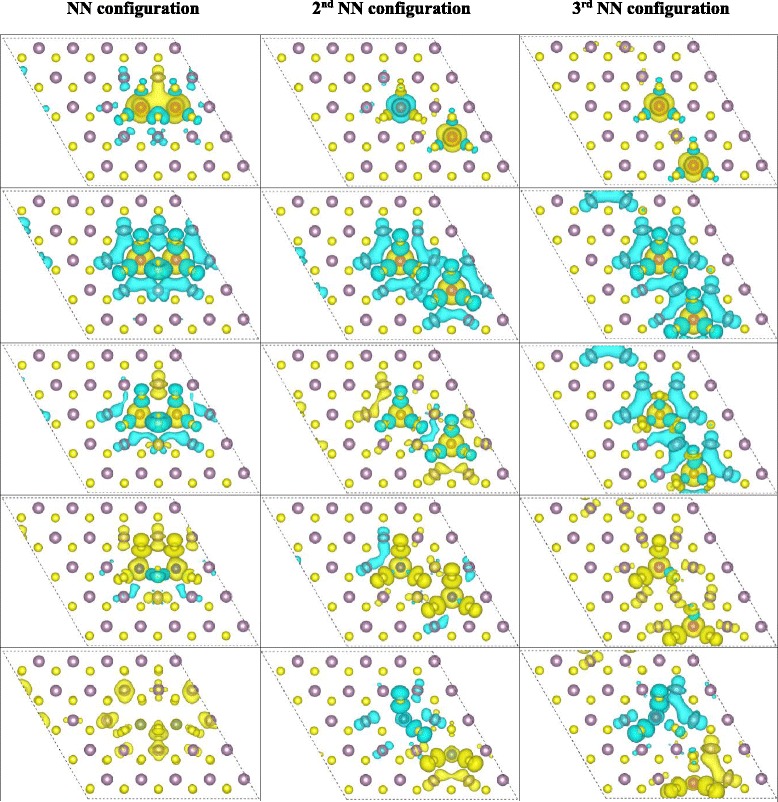
The spin-resolved charge density isosurface (isosurface value at 0.002 e/Å^3^) of TM-doped MoS_2_ monolayer at 8 % impurity concentration. The first, second, third, fourth, and fifth rows are the results for V-, Mn-, Fe-, Co-, and Cu-doped systems, respectively. And the first, second, and third columns correspond to the NN, second NN, and third NN configurations. *Yellow* and *blue colors* represent the spin-up and spin-down charges, respectively

Figure 5 summarized our calculated results on the energy differences between the FM and AFM states for the three configurations of NN, second NN, and third NN. For the four elements of V, Mn, Fe, and Co doping, the energy differences between the FM and AFM states are negative for all the three configurations, which means the FM states are more favorable energetically. Our results on the NN configuration for Mn, Fe, and Co doping agree with previous result [31, 33, 39], and our results on the third configuration agree with Ref [37]. As for Cu doping, the FM states are more favorable for the NN configurations, but for the second NN and third NN configurations, the AFM states are more stable. Table 1 lists the calculated total magnetic moments of the doped system and the local magnetic moments on the impurities for the ground states of the three configurations, and the spin-resolved charge density for the ground states is shown in Fig. 6.

**Table 1:** The magnetic moments (Σμ_i_/μ_total_) for V-, Mn-, Fe-, Co-, and Cu-doped MoS_2_ with impurity concentration at 4, 8, and 12 %

	4 %	8 %			12 %
		NN	2nd NN	3rd NN	NN
V	0.9/1.0	2.2/2.0	1.9/0.0	1.8/2.0	3.6/3.0
Mn	2.9/1.0	5.8/2.0	5.8/2.0	6.4/2.0	6.3/5.0
Fe	3.4/2.0	3.6/2.0	4.4/4.0	6.4/4.0	7.3/2.0
Co	1.1/3.0	3.0/4.0	4.6/6.0	4.3/6.0	0.8/1.0
Cu	0.5/4.9	0.5/3.6	0.6/0.2	0.5/0.0	0.6/3.0

Both the local magnetic moments of the doped atoms (Σμ_i_) and the total magnetic moments of the doped system (μ_total_) are presented. Three configurations with different TM-TM distances were listed for 8 % impurity concentration

As shown in Fig. 6, the spins of the two nearest neighbored dopants are parallel to each other for all the five doped systems. For V, Mn, and Fe doping, the induced spins on the nearby S and Mo atoms are antiparallel to that of the dopants. Thus, the total magnetic moments of the V-, Mn-, and Fe-doped system are smaller than the local magnetic moments on the dopants. As for Co and Cu doping, the total magnetic moments of the doped system are much larger than the local magnetic moments of the impurities because the spin polarizations on the nearby S and Mo atoms are all parallel to that of the dopants. Particularly, the total magnetic moment of the Cu-doped MoS_2_ in NN configuration at impurity concentration of 8 % is 3.6 μ_B_ although the local magnetic moments on the two Cu atoms are only 0.5 μ_B_.

Figure 6 also shows that for V, Mn, Fe, and Co doping in the second NN and third NN configurations, the two dopants are FM coupling or even weakly AFM coupling (the energy difference between the FM and AFM states is 6 meV for V doping in the second configuration). This is similar with the NN configuration. Additionally, the magnetic orderings among the dopants and the nearby host atoms in second and third NN configuration for the four elements are similar with the situation in the NN configuration. In detail, the induced spins on the nearby S and Mo atoms are antiparallel to the impurities for V, Mn, and Fe doping, which leads to the smaller total magnetic moment relative to the local magnetic moments on the dopants, while the FM coupling between the doped Co atoms and the nearby S and Mo atoms makes the total magnetic moment larger than the local ones on the dopants. Moreover, the local magnetic moments of the Fe and Co dopants in second and third NN configurations are larger than those in the NN configuration; thus, the total magnetic moments of second and third NN configurations are larger than those in the NN configuration. For Cu doping in the second and third NN configurations, the AFM states are energetically more stable than the FM states; this is differing from the NN configuration. Figure 6 shows the AFM coupling between the two doped Cu atoms with large separations and the FM exchange with the nearby host S and Mo atoms like the NN configuration. Hence, the total magnetic moments for Cu doping in the second and third NN configurations are very close to 0.

According to our calculations, for the five elements except for Cu doping, the magnetic ordering between the doped atoms and host atoms in the second and third configurations is similar with those in the NN configurations. In contrast, Mishra et al. [39] predicted AFM coupling for the dopants with large separations and FM coupling for the dopants in NN configurations for Fe and Co doping. Additionally, according to Schwigenschlogal et al.’s study [37], Fe and Co doping also lead to AFM ground state in large separations. In this situation, we recalculated the NN, second and third configurations without U parameterizations. The energy differences between the FM and AFM states are summarized in Additional file 1: Figure S2. It shows that for Fe and Co doping in large separations, the AFM states are more favorable energetically, which agrees with previous results [37, 39]. More importantly, we found that the NN configurations are more favorable than the other two configurations with large separations. The total energy of the NN configuration is less than the second and third configurations by 0.2, 0.5, 0.8, 1.0, and 1.3 eV for V, Mn, Fe, Co, and Cu doping, respectively. This is consistent with Liu’s study which shows that the V atoms prefer to stay together in MoS_2_ monolayer.

**Fig. 7:**
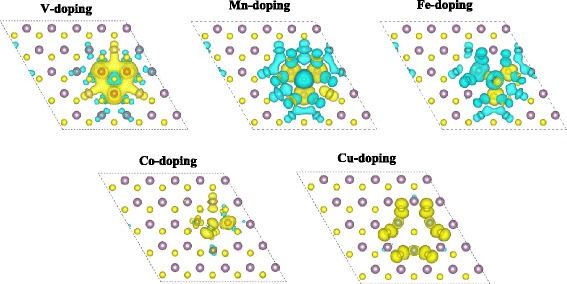
The spin-resolved charge density isosurface (isosurface value at 0.002 e/Å^3^) of TM-doped MoS_2_ monolayer at 12 % impurity concentration. *Yellow* and *blue colors* represent the spin-up and spin-down charges, respectively

**Fig. 8:**
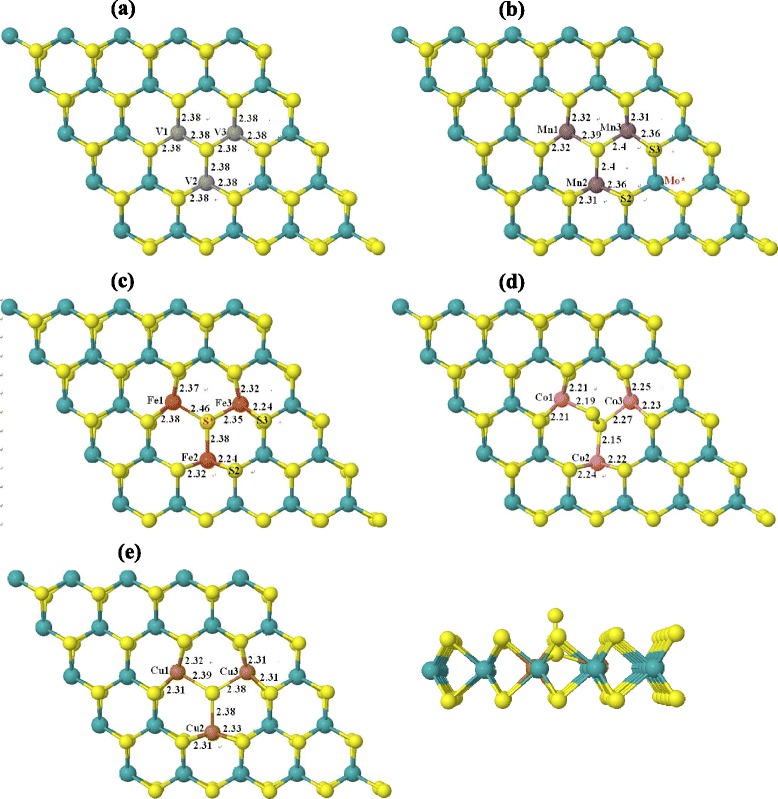
Atomic structures of TM-doped monolayer MoS_2_ at 12 % impurity concentration. **a** V doping. **b** Mn doping. **c** Fe doping. **d** Co doping. **e** Cu doping (top and side views)

On the basis of the study on doping at 8 % impurity concentration, we further studied the TM doping at higher impurity concentration in NN configurations. Three TM atoms replace three nearest neighboring Mo atoms in a 5 × 5 × 1 supercell; the corresponding impurity concentration is 12 %. As shown in Fig. 7, for the five elements except for Fe, the doped TM atoms are FM coupling with each other. For Fe doping, one of the three dopants is AFM coupling with the other two dopants. The reason making the spin polarization of this Fe atom (Fe1) differs from the other two Fe atoms (Fe2 and Fe3) mainly lies in atomic structure. Figure 8 shows the relaxed atomic structures for the five elements doped MoS_2_ monolayer at 12 % impurity concentration. As shown in Fig. 8, the Mn–S bond lengths for the three doped Mn atoms are close, which give rise to the similar spin polarization on the three Mn atoms. The respective local magnetic moments on Mn1, Mn2, and Mn3 are 2.9, 3.1 and 3.1 μ_B_, respectively. Additionally, Fig. 8 shows that the Mn2–S2 and Mn3–S3 bond lengths are little larger than the other Mn–S bond lengths which makes the spin polarization on Mo* atom differs from the other nearby Mo atoms as shown in Fig. 7. As for Fe doping, the Fe–S bond lengths of the Fe1 atom are different from those of the Fe2 and Fe3 atoms. Particularly, the Fe1–S* bond length is larger than the Fe2–S* and Fe3–S* bond lengths, which makes the hybridization of Fe1 3*d* and S* 4*p* differs from the counterparts of Fe2 and Fe3 atoms. Consequently, spin of Fe1 is antiparallel to that of Fe2 and Fe3. More importantly, Fig. 8 shows that the atomic structures of V- and Mn-doped MoS_2_ maintain the original prismatic configuration, but the atomic structures of Co- and Cu-doped MoS_2_ deviate from the prismatic configuration, which is not good for applications in 2D materials.

Figure 7 shows that the magnetic ordering among the dopants and the nearby host atoms at 12 % impurity concentration is similar with the situation at 8 % impurity concentration; for V and Mn (Co and Cu) doping, the induced spins on the nearby host atoms are antiparallel (parallel) to those of the dopants. Thus, the total magnetic moment of V and Mn (Co and Cu) doping is less (larger) than the local magnetic moments of the three dopants. Additionally, our calculations result indicates that for V and Mn doping, the magnetic moments of the doped MoS_2_ increase as the increasing impurity concentration, whereas the magnetisms of Co- and Cu-doped system decrease when impurity concentration increases from 8 to 12 %.

## Conclusions

Our study on MoS_2_ with TM doping at 4 % concentration tells us all the five 3*d* elements of V, Mn, Fe, Co, and Cu doping which give rise to the good diluted magnetic semiconductors. Additionally, we have found that the doped TM atoms prefer to stay in the nearest neighboring positions at high concentrations and couple with each other ferromagnetically. For V, Mn, and Fe doping, the induced spins on the nearby host atoms are antiparallel to that of the impurities, whereas for Co and Cu doping, they are parallel to that of the dopants. It indicates that the local structures around the impurities are deformed from the original prismatic configurations for Co and Cu doping at high impurity concentration although both doping induce strong ferromagnetism into the doped system. Our calculations show that, besides Mn, V is also good candidate to induce and manipulate the magnetism in 2D TMDs.

## Additional files

Additional file 1: Supplemental information. Ferromagnetism in Transitional-Metal Doped MoS_2_ Monolayer. (DOC 228 kb)
