# Supplementary material for: Ferromagnetism in Transitional Metal-Doped MoS2 Monolayer
Source: Nanoscale Res Lett. 2016 Mar 22;11:154. doi: 10.1186/s11671-016-1376-y (PMC4801828; doi:10.1186/s11671-016-1376-y)
Supplement: Additional file 1: — Supplemental information. Ferromagnetism in Transitional-Metal Doped MoS2 Monolayer. (DOC 228 kb) [file 11671_2016_1376_MOESM1_ESM.doc]

**Supplemental Information**

**Ferromagnetism in Transitional-Metal Doped MoS_2_ Monolayer**

*Xiao-Li Fan^1,2*^, Yu-Rong An^1^, Wen-Jun Guo^1^*

^1^ State Key Laboratory of Solidification Processing, School of Material Science and Engineering, Northwestern Polytechnical University, 127 YouYi Western Road, Xi’an, Shaanxi 710072, China

^2^Beijing Computational Science Research Center, Beijing, 100094, China

*[xlfan@nwpu.edu.cn](mailto:xlfan@nwpu.edu.cn)

**
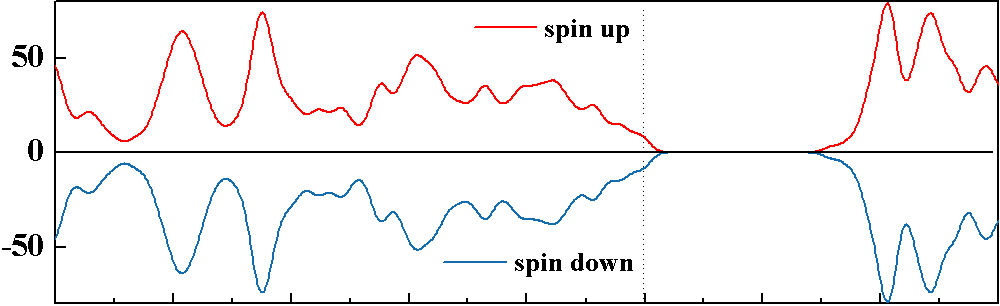
**

**(a)**

**
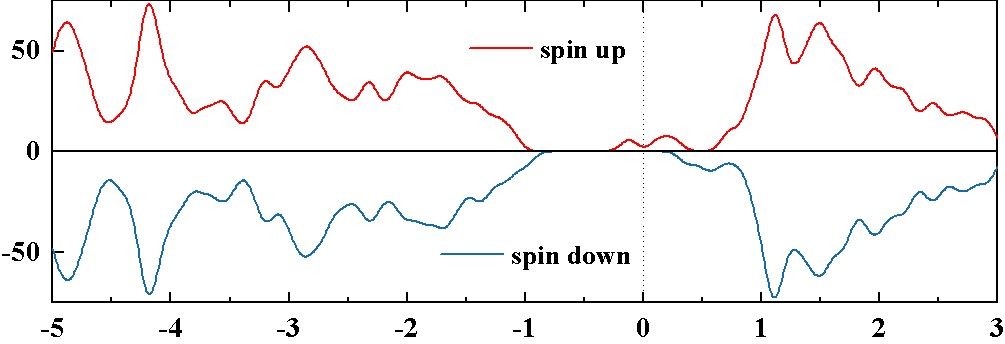
**

**(b)**

**
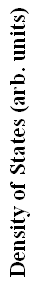
**

**(c)**

**
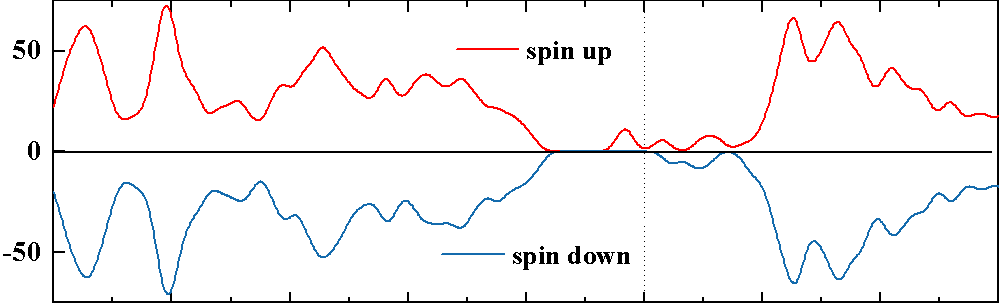
**

**
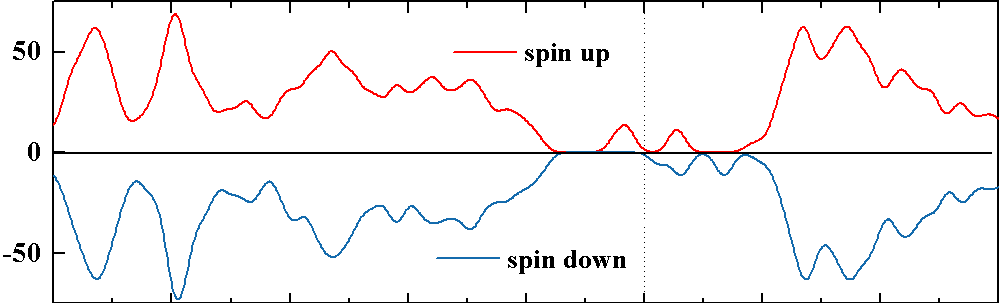
**

**(d)**

**
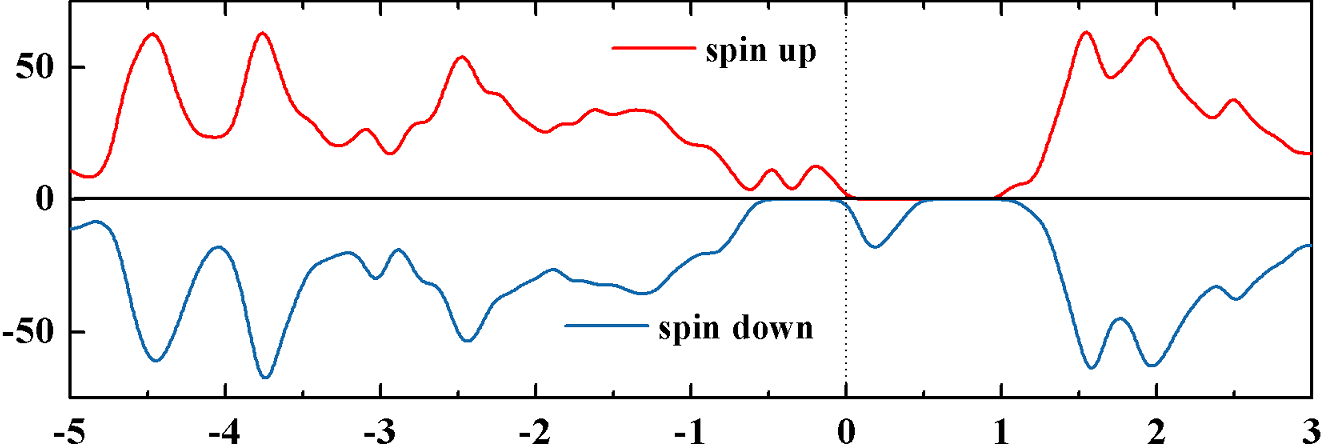
**

**(e)**

**
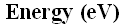
**

**Figure S1** Density of states of monolayer MoS_2_ with V, Mn, Fe, Co, Cu doping at 4% impurity concentration. (a) V doping,（b）Mn doping, (c) Fe doping, (d) Co doping, (e) Cu doping.

**
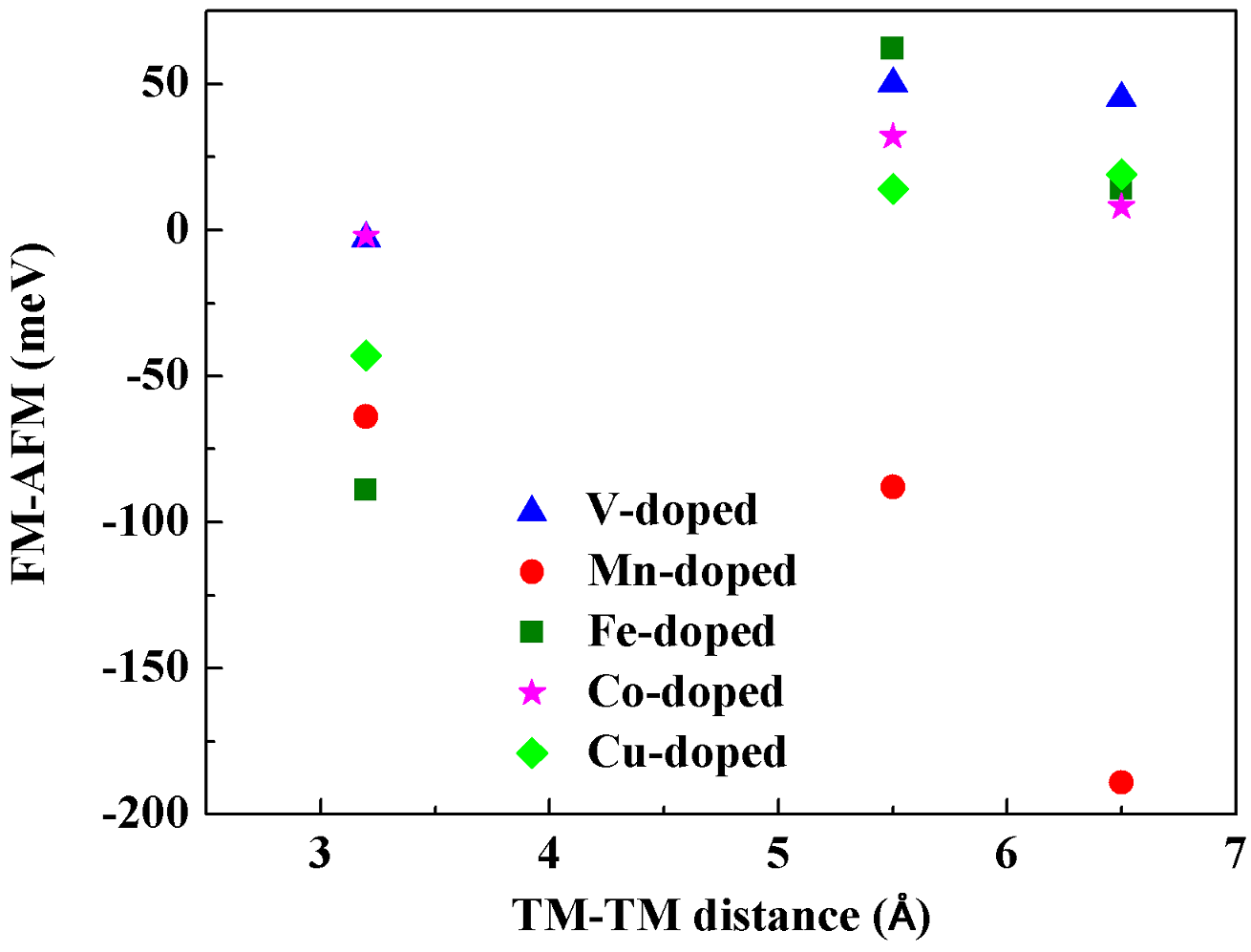
**

**Figure S2** Energy differences of the FM ordering over AFM ordering for V, Mn, Fe, Co and Cu doped MoS_2_ as a function of the distance between the two doped TM atoms. The corresponding impurity concentration is 8%.
